# Addressing Inequity in Spatial Access to Lung Cancer Screening

**DOI:** 10.3390/curroncol30090586

**Published:** 2023-08-31

**Authors:** Jonathan Simkin, Edwin Khoo, Maryam Darvishian, Janette Sam, Parveen Bhatti, Stephen Lam, Ryan R. Woods

**Affiliations:** 1 BC Cancer, Provincial Health Services Authority, Vancouver, BC V5Z 4C2, Canada; 2 BC Cancer Screening, BC Cancer, Provincial Health Services Authority, Vancouver, BC V5Z 1G1, Canada; edwin.khoo@bccancer.bc.ca (E.K.); maryam.darvishian@ubc.ca (M.D.); jsam@bccancer.bc.ca (J.S.); slam2@bccancer.bc.ca (S.L.); 3 Cancer Control Research, BC Cancer Research Institute, Vancouver, BC V5Z 1G1, Canada; pbhatti@bccrc.ca (P.B.); rwoods@bccancer.bc.ca (R.R.W.); 4 School of Population and Public Health, Faculty of Medicine, University of British Columbia, Vancouver, BC V6T 1Z3, Canada; 5 Faculty of Health Sciences, Simon Fraser University, Burnaby, BC V5A 1S6, Canada

**Keywords:** access, geospatial, health equity, health service access, lung cancer, prevention, cancer screening

## Abstract

Background: The successful implementation of an equitable lung cancer screening program requires consideration of factors that influence accessibility to screening services. Methods: Using lung cancer cases in British Columbia (BC), Canada, as a proxy for a screen-eligible population, spatial access to 36 screening sites was examined using geospatial mapping and vehicle travel time from residential postal code at diagnosis to the nearest site. The impact of urbanization and Statistics Canada’s Canadian Index of Multiple Deprivation were examined. Results: Median travel time to the nearest screening site was 11.7 min (interquartile range 6.2–23.2 min). Urbanization was significantly associated with shorter drive time (*p* < 0.001). Ninety-nine percent of patients with ≥60 min drive times lived in rural areas. Drive times were associated with sex, ethnocultural composition, situational vulnerability, economic dependency, and residential instability. For example, the percentage of cases with drive times ≥60 min among the least deprived situational vulnerability group was 4.7% versus 44.4% in the most deprived group. Conclusions: Populations at risk in rural and remote regions may face more challenges accessing screening services due to increased travel times. Drive times increased with increasing sociodemographic and economic deprivations highlighting groups that may require support to ensure equitable access to lung cancer screening.

## 1. Introduction

Lung cancer is the most commonly diagnosed cancer and leading cause of cancer death in Canada [1] and worldwide [2]. In 2021, an estimated 29,600 Canadians were diagnosed with lung cancer and an estimated 21,000 died from this disease [1]. At 22%, the five-year net survival for lung cancer is among the lowest of all types of cancer [1]. Lung cancer screening with low dose computed tomography (LDCT) has been shown to significantly reduce lung cancer mortality. Three randomized clinical trials showed a 20% to 39% mortality reduction benefit of lung screening using LDCT versus chest X-ray or usual care [3,4,5]. Despite evidence supporting lung screening through LDCT and coverage by Medicare and most commercial insurers, screening uptake remains uneven and slow in the United States (US) [6].

An important issue in lung screening is addressing inequity by improving access to screening services to Indigenous peoples, socioeconomically deprived population groups, and those living in rural areas because of existing inequities [7,8,9,10,11]. For example, negative gradients in lung cancer risk for individual-level socioeconomic status have been reported in both Canada and the United States (US) [7,8,11]. Geographic variation has been reported at both national and provincial levels [1,9,11]. In Canada, First Nations people had lower survival for cancers of the lung and bronchus compared to non-First Nations people [10]. Differences in survival are related to stage of diagnosis, emphasizing the importance of access to screening to improve health outcomes. For example, in Canada, the three-year net survival for lung cancer diagnosed at stage 4 was 5% compared to 71% for lung cancer diagnosed at stage 1 [12]. In the US, studies suggest that while most of the screen-eligible population are within close proximity to a screening site, a small proportion has poor spatial access [13,14,15]. In Canada, increasing travel time and level of urbanization was associated with lower screening rates in breast mammography screening [16,17], and colorectal cancer screening [18,19,20,21].

To our knowledge, no studies have yet examined spatial access to fixed LDCT lung cancer screening sites by neighborhood measures of socioeconomic deprivation and few at the level of urbanization [15]. To address this gap in knowledge, we used a population-based cohort of lung cancer cases in British Columbia, Canada, as a proxy for the screen-eligible population and examined travel time, as an indicator of spatial access, to fixed lung cancer screening sites. Although eligibility for lung screening is driven by age and smoking history at present, targeting screening services to areas in relation to lung cancer incidence as a proxy may be an effective way to improve lung cancer outcomes. This is especially the case when accurate smoking history and other lung cancer risk factors used in a risk prediction tool, such as the PLCOm2012, are not available.

In Canada, health services are delivered at the provincial or territory level. British Columbia (BC) is Canada’s third largest province with an area approximately two times the size of the State of California in the United States. It is populated by over 5.1 million people [22]. BC health services are planned and delivered at various health administrative areas, with the largest consisting of five regional health authorities (HAs). Nested within HAs are 16 Health Service Delivery Areas (HSDA), 89 Local Health areas, and 218 Community Health Service Areas (CHSA) [23]. BC Cancer routinely reports screening indicators at HSDA regional levels. The CHSA health boundary was introduced by the Ministry of Health in 2019 to provide a standard geographic definition for community level analyses for health service planning [23].

We hypothesize that the majority of the screen-eligible population are within close proximity to a screening site. However, the minority with poor spatial access will also demonstrate greater socioeconomic deprivation.

## 2. Materials and Methods

### 2.1. Study Population and Setting

This study was approved by the University of British Columbia–BC Cancer Research Ethics Board.

Lung screening programs are slowly being implemented across Canada. British Columbia (BC) implemented an organized lung cancer screening program, using low-dose computed tomography (CT) scans that are carried out through fixed sites, in 2022. All British Columbians that are 55 to 74 years of age, current or former smokers, who smoked for at least 20 years with a six-years lung cancer risk >1.5% using the PLCOm2012 risk prediction tool [24] are eligible to participate in the provincial lung cancer screening program.

This was a retrospective population-based descriptive study. A total of 36 lung cancer screening sites around the province with the necessary CT facilities have been identified. As a proxy for the potential screen eligible population, we identified all newly diagnosed (incident) lung cancer cases in BC between 2015 and 2019 aged 55 to 80 years (*N* = 12,886) using the BC Cancer Registry. Cases up to 80 years were included in the study cohort to include age ranges for both Canadian and US recommendations. For each case, we obtained information on sex, age at diagnosis, histologic subtype, tumor stage, and complete residential postal-code at the time of diagnosis. In Canada, six-digit postal codes typically represent one side of a city block in urban areas, while rural area postal codes are larger.

### 2.2. Geocoding

The Statistics Canada Postal Code Conversion File Plus (PCCF+) was used to assign spatial locations (i.e., longitude-latitude coordinates) to cases from the complete postal codes captured by the cancer registry. Records with non-valid or missing postal codes were excluded (*N* = 198). Records were linked from spatial coordinates to the BC CHSA boundary map to determine BC administrative health areas (CHSA and HSDA areas) at diagnosis and the BC CHSA Urban-Rural Classification [23]. The CHSA Urban-Rural classification includes seven categories: Metropolitan, Large Urban, Medium Urban, Small Urban, Rural Hub, Rural, and Remote. These categories are generated by the BC Ministry of Health and derived using two Statistics Canada Census metrics: Population Centre and Rural Area Classification and Index of Remoteness. CT screening sites (*N* = 36) were provided by the BC Lung Screening Program and latitude and longitude coordinates for these sites were obtained using a standardized BC healthcare facility geolocating reference file [25]. The Statistics Canada Canadian Index of Multiple Deprivation [26] (CIMD) was linked to the cases at the government census dissemination area level (DA), which is reflective of the neighborhood level. CIMD data are census-derived and geographically indexed variables that enable analyses on sociodemographic and economic inequalities. They are publicly available by Statistics Canada with the intent to support policy planning and evaluation, research and analysis and resource allocation [26].

The BC-CIMD dataset [26] used in this analysis contains four neighborhood-level dimensions, each composed of various census measures: residential instability, economic dependency, ethnocultural composition and situational vulnerability (Supplementary Table S1). The CIMD variables were available in factor scores and quintiles. In this study, we use quintile rankings. Quintiles were derived by ordering factor scores for neighborhoods (*N* = 7617) from smallest to largest and then divided into quintiles categorized from 1 through 5. A value of 1 corresponds to neighborhoods that were the least deprived for that dimension, and a value of 5 corresponds to neighborhoods that were the most deprived. For ethnocultural composition, a value of 5 corresponds to the highest level of ethnocultural composition.

### 2.3. Travel Time

The outcome variable of interest was the shortest travel time, in minutes, by car from the location associated with a patient’s residential postal code at diagnosis to the nearest screening site. Travel time was calculated using the Open Source Routing Machine (OSRM) (Version: 5.27.1, The Free DSB Project, Vancouver BC, Canada) [27] and the OSRM R package (Version: 4.1.1, Centre National de la Recherche Scientifique, Paris, France) [28].

### 2.4. Statistical Analysis

Travel time was treated as both a continuous and categorical variable. We reported the median and interquartile ranges for travel time to the nearest CT center in minutes. Drive time categories were as follows: <20 min, 20–<40 min, 40–<60 min and ≥60 min. These categories were informed by those used in previous travel time studies [29,30].

A bivariate analysis of travel time categories with each variable of interest was conducted including: age, sex, histologic type, stage at diagnosis, level of urbanization and the four CIMD variables. A chi-square test of independence was used to assess associations between travel time and each variable of interest. A *p*-value ≤ 0.05 was considered statistically significant.

Geographic variation in drive time was assessed at the HSDA-level with box plots to provide high-level distributions of drive time among these relatively large health areas. Community level variation was explored by mapping median travel time at the CHSA-level. Using the Monte Carlo simulation method [31] (*N* = 999 simulations), a global Moran’s I statistic was calculated to test whether or not median drive times were clustered.

## 3. Results

Overall, 51.6% of lung cancer patients were female (*N* = 6553), and the median age at time of diagnosis was 70 years (interquartile range (IQR) = 64 to 75 years) (Table 1). The most common histologic type was non-small cell lung cancer (80.3%, *N* = 10,194). The lung cancers were most commonly diagnosed at stage IV (43.7%, *N* = 5539). Most patients resided in Metropolitan areas (36.0%, *N* = 4573), followed by Medium Urban (18.4%, *N* = 2332) and Rural areas (14.8%, *N* = 1884) (Table 1). When examining CIMD factors, the patients generally resided in more deprived areas with low ethnocultural composition, greater residential instability and greater economic dependency (Table 1).

The median travel time to the nearest screening site was 11.7 min (Interquartile Range (IQR)= 6.2 to 23.2 min). Overall, 69.8% of cases (*N* = 8856) were less than 20 min from a screening site. In contrast, 8.0% of cases (*N* = 1014) were over 60 min from a screening site (Table 2).

In general, male sex, lower neighborhood urbanization, lower neighborhood ethnocultural composition, lower neighborhood residential instability, greater neighborhood situational vulnerability and greater neighborhood economic dependency were associated with increased drive times (*p* < 0.05) (Table 3).

Although cases that were 60 min or greater from a screening site only represented 8.0% of all cases (Table 2), 44.2% resided in neighborhoods with the highest situational vulnerability, 50.0% in the lowest ethnocultural composition, 5.3% in the highest residential instability, and 55.7% in the highest economic dependency (Table 3). In comparison, cases that were <20 min from a screening site represented 69.8% of all cases (Table 2). Among these cases, 22.0% resided in neighborhoods with the highest situational vulnerability, 14.6% in the lowest ethnocultural composition, 31.9% in the highest residential instability, and 22.1% in the highest economic dependency (Table 3).

**Figure 1:**
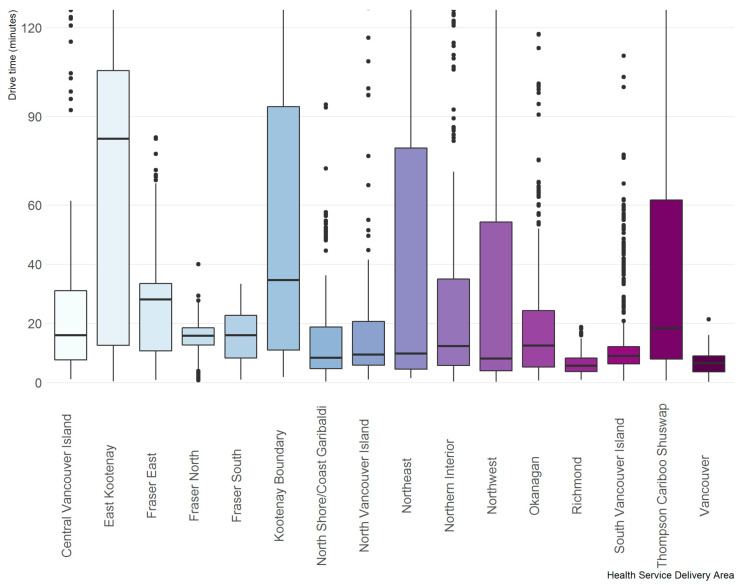
Drive time distribution by health service delivery area. The y-axis was restricted to a maximum of 120 min to better visualize drive times across all HSDA areas. There were 338 records of drive times greater than 120 min (2.6% of the analytic cohort).

Drive time distributions across HSDAs regions are provided in Figure 1. Generally, median travel times were less than 20 min across HSDAs. Some HSDA regions showed narrow distributions, such as Vancouver, Richmond, Fraser North and Fraser South. In contrast, East Kootenay, Kootenay Boundary, Northeast, among others, showed wide distributions. Most cases in Fraser East were within 40 min of a screening site. East Kootenay and Kootenay Boundary HSDAs showed most cases within 20 min or over 60 min from a screening site (Figure 1).

**Figure 2:**
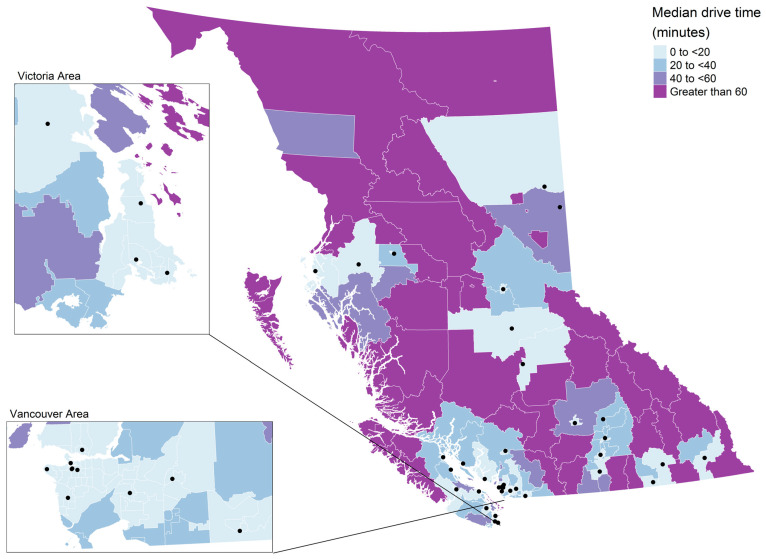
Median drive time to the closest screening site by community health service area. Lung screening sites are represented by black dots.

**Figure 3:**
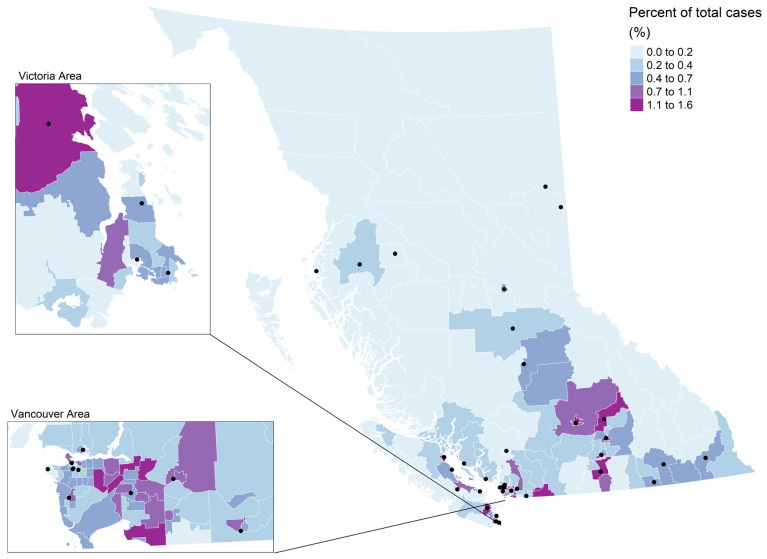
Proportion of lung cancer cases by community health service area. Lung screening sites are represented by black dots.

A choropleth map of median drive time by CHSA is shown in Figure 2. The Moran’s I statistic for median drive times was 0.47 (*p* = 0.002) indicating significant clustering. There were 118 CHSAs (54.1%) with a median drive time less than 20 min. Short drive times were generally located in the southwest mainland and island CHSAs and some CHSAs in the southern and central interior (Figure 2). CHSAs with a median drive time less than 20 min represented 70.4% (*N* = 8937) of lung cancer cases in BC (Figure 3). There were 47 CHSAs (21.6%) with a median drive over 60 min. These were generally located in the southeastern CHSAs, as well as central and northern CHSAs (Figure 3). CHSAs with drive times greater than 60 min represented 6.8% of lung cancer cases in BC (Figure 3). A choropleth map of the CHSA areas by level of urbanization was provided in Supplemental Figure S1.

## 4. Discussion

To our knowledge, this is the first study to examine spatial access to lung screening sites in a regional health care setting considering the impact of age, sex, level of urbanization, and multiple indices of deprivation. While the bulk of proxy, screen-eligible participants (69.8%) were less than 20 min from a screening site, our results do highlight important differences in spatial access to screening sites among rural versus urban participants and those with greater levels of sociodemographic and economic deprivation.

Cases that were 60 min or greater from a screening site (i.e., poor spatial access) represented a small proportion of participants (8.0%, *N* = 1014). Relative to cases with short spatial access (i.e., <20 min from a screening site), cases with poor spatial access were more likely to be male (51.0% vs. 48.6%) and reside in rural areas (90.4% vs. <0.5%). They also were more likely to reside in neighborhoods with lower ethnocultural composition (50.0% vs. 14.6%), greater situational deprivation (44.2% vs. 22.0%), economic dependence (55.7% vs. 22.1%), and lower residential instability (5.3% vs. 31.9%).

Consistent with our findings, studies in the US have reported that most of the target population are within close proximity to a lung screening site. Tailor et al. reported that over 80% of people who smoked in the US were within 15 miles of a CT facility [13]. Sahar et al. reported that most people 55–79 years of age in over 63% of US counties were within 40 miles of a screening site [14]. However, almost all people 55–79 years of age in approximately 6% of US counties were found to be farther than 40 miles from a screening site [14]. This figure increased to 16% when a 20-mile threshold was considered [14]. Importantly, a larger proportion of those with limited access to a screening site are rural [15]. Populations with low spatial access are less likely to participate in screening services. In Canada, increased travel time has consistently been associated with lower participation in existing screening services [16,17] and radiotherapy (RT) utilization [18,29,32,33,34].

Few previous studies have examined socioeconomic characteristics of areas with poor spatial access, which is an important consideration for program planning and implementation. In the US, counties with poor spatial access to a lung screening site were generally low resource areas with high lung cancer mortality rates [30]. Importantly, less affluent US counties generally experience a disproportionate burden of preventable cancers, e.g., lung cancer [35]. Similar disparities in lung cancer risk across socioeconomic groups have been reported in Canada [7,36].

As in our study, decreasing population density and rurality in the US has been associated with lower spatial access to a lung screening site [13,15]. Importantly, smoking prevalence and disease burden also vary across levels of urbanization [37]. In BC, smoking prevalence is generally higher in rural areas [37]. Lung cancer risk also varies geographically and has been shown to be elevated in rural versus urban population, such as BC’s Northern and Interior health administrative regions [38,39]. Across Canada, rural populations had higher age-standardized lung cancer incidence and mortality rates, as well as advanced stage specific rates, compared to urban populations [18]. Lung cancer incidence and mortality were also greater in rural compared to urban populations in the US [40]. Overall, spatial access should be considered along with socioeconomic and demographic factors, smoking prevalence, and lung cancer mortality to maximize the impact of lung screening programs.

Despite high overall spatial access in the US, the proportion of lung screen-eligible people that have undergone screening is relatively low in comparison to other screening programs. From 2010 to 2015, the figure was reported as 3.9% [41] and in 2017 it was reported as 14.4% [42]. As such, screening participation is likely influenced by a combination of factors at the patient-level including awareness, beliefs and attitudes about lung screening, stigmatization of smoking, socioeconomic status and sociocultural factors [43,44,45]. Factors related to primary care may also contribute, including provider gaps in knowledge about screening guidelines and tools to support patient-provider discussions about the benefits and harms of screening [46].

Targeting screening services to areas in relation to lung cancer incidence as a proxy may be an effective way to improve lung cancer outcomes when data on risk factors used in risk prediction tools are not available. Sahar et al. also recommended integrating regional measures of the lung cancer burden, such as lung cancer mortality rates, to guide lung screening interventions [15]. Screening eligibility criteria is an evolving science that will change as an increasing number of lung cancers will be arising from those who have never smoked or have a modest smoking history [47,48]. Targeting lung cancer screening services to areas where lung cancer patients are found may be more effective than smoking history in the long-term.

### Study Limitations

Firstly, while our analyses did benefit from the availability of CMID measures, these were assessed at the neighborhood level and may not reflect individual-level characteristics. Secondly, this study focused on descriptive analyses to quantify spatial access and associated factors. The results do not account for potential confounding and additional work is required to explore and quantify these relationships further while adjusting for confounding.

Thirdly, the scope of this study encompassed a universal health care context and was conducted within the framework of a province-wide organized screening program. While these findings may reasonably be extended to other Canadian provinces with similar organized screening programs, it is essential to consider the influence of varying health care systems, population demographics, and cancer screening delivery models. It is worth noting that our results align with studies conducted in the US [13,15], where evidence suggests a commendable coverage for lung screening on a broader scale, yet spatial access disparities persist among certain subpopulations. This alignment supports the notion that our findings hold relevance beyond national boundaries. Nevertheless, the extent to which these findings can be generalized to diverse regions hinges on factors such as population dispersion and concentration. In regions similar to that of British Columbia, where populations are dispersed across a large land mass with few high-density metropolitan areas, our findings could likely apply. Finally, in this study, a complete six-digit postal code was assigned to a geocoordinate using the Statistics Canada PCCF+ program. PCCF+ uses population weighting and random allocation to inform the geocoordinate of postal codes, specifically when postal codes match to the multiple potential reference points. Positional accuracy is better among urban versus rural areas [49,50] and therefore, drive times are likely more precise for urban versus rural individuals. We would expect positional accuracy to be the least precise for rural individuals that are 60+ min from a CT site. The magnitude of this positional error (distance error between PCCF+ geocoordinates and true reference points) is unknown, however, based on prior literature [49,50] it is typically small. For example, according to Khan et al., most urban postal codes link to a single blockface (i.e., one side of a street) or dissemination block (i.e., an area bounded on all sides by roads) and this represents nearly 75% of the population [49]. The other postal codes match to multiple representative points and the PCCF+ geocodes these postal codes randomly using population weights [51]. From their study, the percentages of the sample geocoded to within 500 m of their full street address were 56% for small communities of 10,000 to 99,999 population and more than 70% for communities of at least 500,000 [49]. Further, we expect the error would be amplified on a finer scale of drive time as opposed to our drive time categories.

Our study provides critical insights on spatial disparities in access to lung cancer screening services. Screening programs can expand screening sites strategically in underserved areas for equitable access to improve access. By pinpointing regions with poor spatial access, screening programs can allocate resources more efficiently to reach those who need it the most. Additionally, using lung cancer incidence as a proxy for the screen-eligible population, in lieu of comprehensive smoking prevalence data, can guide resource allocation for screening and health promotion efforts. Prioritizing high-risk areas, indicated by our method, can improve resource allocation and help early detection, reduce mortality, and community health. Lastly, as lung cancer screening programs are introduced in various Canadian provinces, our study underscores the importance of a strong foundational understanding of spatial access. Programs can adopt a similar methodology, utilizing open source and census-related data to identify communities facing access challenges.

While LDCT screening is inherently conducted through fixed sites requiring in-person attendance, it is important to acknowledge the potential role of telehealth in enhancing various aspects of the screening process and advancing patient care. For example, BC Cancer Lung Screening has virtual care options for individuals without a primary care physician to support eligible participants in the referral process for LDCT lung screening [52]. Notably, Magarinos et al. showcased the viability of single-encounter telemedicine lung cancer screening [53]. This approach involves leveraging telemedicine following an individual undergoing LDCT at a facility. Through telemedicine, discussions of results, coordination of follow-up procedures, and facilitation of smoking cessation counseling were effectively managed through telemedicine, thereby emphasizing the multifaceted advantages of telehealth within the broader framework of lung cancer screening.

In conclusion, we found that most of our study population were within 20 min from a lung screening site. However, spatial access differed geographically and was associated with deprivation across some sociodemographic and economic measures, highlighting populations that may require additional support to ensure equitable access to lung cancer screening services.

## Figures and Tables

**Table 1:** Descriptive statistics on the study cohort and variables of interest such as age, sex, year of diagnosis, histology, stage at diagnosis, level of urbanization and measures of deprivation, British Columbia, 2015–2019.

Variable	Total (N) *N* = 12,688	Proportion (%)
Age (years)		
Median [IQR]	70 (64–75)	
Age groups (years)		
55–59	1211	9.5
60–64	2096	16.5
65–69	2722	21.5
70–74	3168	25
75–80	3491	27.5
Sex		
Females	6553	51.6
Males	6135	48.4
Year of diagnosis		
2015	2472	19.5
2016	2400	18.9
2017	2528	19.9
2018	2567	20.2
2019	2721	21.4
Histology type		
Non-small cell lung cancer	10,194	80.3
Small cell lung cancer	1226	9.7
Sarcomas and other specified malignant neoplasms	15	0.1
Unspecified	1253	9.9
Stage ^1^		
I	2602	20.5
II	979	7.7
III	2587	20.4
IV	5539	43.7
Unknown	961	7.6
Occult	20	0.2
CHSA urban-rural classifications		
Metropolitan	4573	36
Large Urban	1675	13.2
Medium Urban	2332	18.4
Small Urban	1223	9.6
Rural Hub	896	7.1
Rural	1884	14.8
Remote	105	0.8
CIMD variables		
Ethnocultural composition ^1^		
Q1	2687	21.2
Q2	3114	24.5
Q3	2781	21.9
Q4	2218	17.5
Q5	1869	14.7
Missing	19	0.1
Situational vulnerability ^2^		
Q1	2250	17.7
Q2	2479	19.5
Q3	2444	19.3
Q4	2629	20.7
Q5	2867	22.6
Missing	19	0.1
Residential instability ^2^		
Q1	1935	15.3
Q2	2350	18.5
Q3	2482	19.6
Q4	2663	21
Q5	3239	25.5
Missing	19	0.1
Economic dependency ^2^		
Q1	1858	14.6
Q2	2200	17.3
Q3	2375	18.7
Q4	2506	19.8
Q5	3730	29.4
Missing	19	0.1

^1^ For ethnocultural composition, a value of 5 corresponds to the greatest ethnocultural composition. ^2^ A value of 1 corresponds to the least deprived for that dimension, and a value of 5 corresponds to the most deprived.

**Table 2:** Descriptive statistics on travel time (in minutes) from residential postal code to CT sites, British Columbia, 2015–2019.

Variable	Total (*N*) *N* = 12,688	Proportion (%)
Travel time (minutes)		
Median [IQR]	11.7 [6.2–23.2]	
Travel time categories (minutes)		
<20	8856	69.8
20 to 40	2246	17.7
40 to 60	572	4.5
60+	1014	8.0

**Table 3:** Bivariate analysis (chi-square test of independence) between drive time (minutes) and variables of interest such as age, sex, stage at diagnosis, level of urbanization, and measures of deprivation, British Columbia, 2015–2019.

	Drive Time Categories				*p* Value
Variable	<20	20–40	40–60	60+	
Age					0.37
55–59	852 (9.6)	208 (9.3)	55 (9.6)	96 (9.5)	
60–64	1464 (16.5)	361 (16.1)	86 (15.0)	185 (18.2)	
65–69	1894 (21.4)	457 (20.3)	137 (24.0)	234 (23.1)	
70–74	2198 (24.8)	585 (26.0)	134 (23.4)	251 (24.8)	
75+	2448 (27.6)	635 (28.3)	160 (28.0)	248 (24.5)	
Sex					0.018
Females	4555 (51.4)	1218 (54.2)	283 (49.5)	497 (49)	
Males	4301 (48.6)	1028 (45.8)	289 (50.5)	517 (51)	
Stage					0.28
I	1847 (22.6)	454 (21.8)	95 (18.1)	206 (22)	
II	677 (8.3)	189 (9.1)	45 (8.6)	68 (7.3)	
III	1792 (22)	466 (22.3)	112 (21.3)	217 (23.2)	
IV	3843 (47.1)	978 (46.9)	273 (52)	445 (47.5)	
Level of urbanization					<0.001
Metropolitan	4204 (47.5)	368 (16.4)	<5 (0.9)	<5 (0.5)	
Large Urban	1616 (18.2)	<60 (2.7)	<5 (0.9)	<5 (0.5)	
Medium Urban	1704 (19.2)	600 (26.7)	<30 (5.2)	<5 (0.5)	
Small Urban	757 (8.5)	367 (16.3)	<100 (17.5)	<5 (0.5)	
Rural Hub	124 (1.4)	297 (13.2)	171 (29.9)	304 (30)	
Rural	445 (5)	551 (24.5)	276 (48.3)	612 (60.4)	
Remote	6 (0.1)	<5 (0.2)	<5(0.9)	<5 (0.5)	
CIMD variables					
Ethnocultural composition *					
Q1	1289 (14.6)	693 (30.9)	200 (35.4)	505 (50.0)	<0.001
Q2	1796 (20.3)	712 (31.8)	242 (42.8)	364 (36.0)	
Q3	2011 (22.7)	553 (24.7)	100 (17.7)	117 (11.6)	
Q4	1965 (22.2)	205 (9.1)	23 (4.1)	25 (2.5)	
Q5	1791 (20.2)	78 (3.5)	0 (0)	0 (0)	
Situational vulnerability *					<0.001
Q1	1601 (18.1)	525 (23.4)	76 (13.5)	48 (4.7)	
Q2	1755 (19.8)	491 (21.9)	117 (20.7)	116 (11.5)	
Q3	1714 (19.4)	445 (19.9)	124 (21.9)	161 (15.9)	
Q4	1836 (20.7)	423 (18.9)	131 (23.2)	239 (23.6)	
Q5	1946 (22.0)	357 (15.9)	117 (20.7)	447 (44.2)	
Residential instability *					<0.001
Q1	1231 (13.9)	460 (20.5)	76 (13.5)	168 (16.6)	
Q2	1385 (15.6)	551 (24.6)	156 (27.6)	258 (25.5)	
Q3	1509 (17.0)	467 (20.8)	214 (37.9)	292 (28.9)	
Q4	1902 (21.5)	427 (19.1)	95 (16.8)	239 (23.6)	
Q5	2825 (31.9)	336 (15)	24 (4.2)	54 (5.3)	
Economic dependency *					<0.001
Q1	1477 (16.7)	225 (10)	63 (11.2)	93 (9.2)	
Q2	1763 (19.9)	320 (14.3)	37 (6.5)	80 (7.9)	
Q3	1818 (20.5)	372 (16.6)	74 (13.1)	111 (11)	
Q4	1834 (20.7)	417 (18.6)	91 (16.1)	164 (16.2)	
Q5	1960 (22.1)	907 (40.5)	300 (53.1)	563 (55.7)	

* A value of Q1 corresponds to neighborhoods that were the least deprived for that dimension, and a value of Q5 corresponds to neighborhoods that were the most deprived. For ethnocultural composition, a value of Q5 corresponds to a neighborhood with the greatest ethnocultural composition.

## Data Availability

The dataset used and analyzed during the current study were obtained from the BC Cancer Registry and are not publicly available due to privacy legislation and institutional data sharing agreements. Data however can be requested through a data access request to BC Cancer following their processes at http://www.bccancer.bc.ca/health-professionals/professional-resources/bc-cancer-registry/request-registry-data (accessed on 1 September 2021).

## Supplementary Materials

The following supporting information can be downloaded at: https://www.mdpi.com/article/10.3390/curroncol30090586/s1, Figure S1: A choropleth map of community health service areas by level of urbanization. Lung screening sites are represented by black dots; Table S1: Description of CIMD Variables and corresponding index indicators.
