# Supplementary material for: Addressing Inequity in Spatial Access to Lung Cancer Screening
Source: Curr Oncol. 2023 Aug 31;30(9):8078–91. doi: 10.3390/curroncol30090586 (PMC10529474; doi:10.3390/curroncol30090586)
Supplement: Supplementary file 1 [file curroncol-30-00586-s001.zip › Table S1.pdf]

Supplementary Table S1. Description of CIMD Variables and corresponding index indicators.

Variable	Description	Indicators
Ethno-Cultural Composition	Refers to the community make-up of immigrant populations.	• Proportion of population who self-identify as visible minority<br>• Proportion of population that is foreign-born<br>• Proportion of population with no knowledge of either official language (linguistic isolation)<br>• Proportion of population who are recent immigrants (arrived in five years prior to Census)
Situational Vulnerability	Refers to variations in socio-demographic conditions in the areas of housing and education, while taking into account other demographic characteristics.	• Proportion of population that identifies as Indigenous<br>• Proportion of population nagged 25-64 without a high school diploma<br>• Proportion of dwellings needing major repairs<br>• Proportion of population that is low-income<br>• Proportion of single parent families
Economic Dependency	Refers to reliance on the workforce, or a dependence on sources of income other than employment income.	• Proportion of population participating in labour force (aged 15 and older)<br>• Proportion of population aged 65 and older<br>• Ratio of employment to population<br>• Dependency ratio (population aged 0-14 and aged 65 and older divided by population aged 15-62)
Residential instability	Refers to the tendency of neighbourhood inhabitants to fluctuate over time, taking into consideration both housing and familial characteristics.	• Proportion of dwellings that are apartment buildings<br>• Proportion of persons living alone<br>• Proportion of dwellings that are owned<br>• Proportion of the population who moved within the past five years

Information in this table were sourced from The Canadian Index of Multiple Deprivation User Guide.
