# Supplementary figures and images for: Addressing Inequity in Spatial Access to Lung Cancer Screening
Source: Curr Oncol. 2023 Aug 31;30(9):8078–91. doi: 10.3390/curroncol30090586 (PMC10529474; doi:10.3390/curroncol30090586)

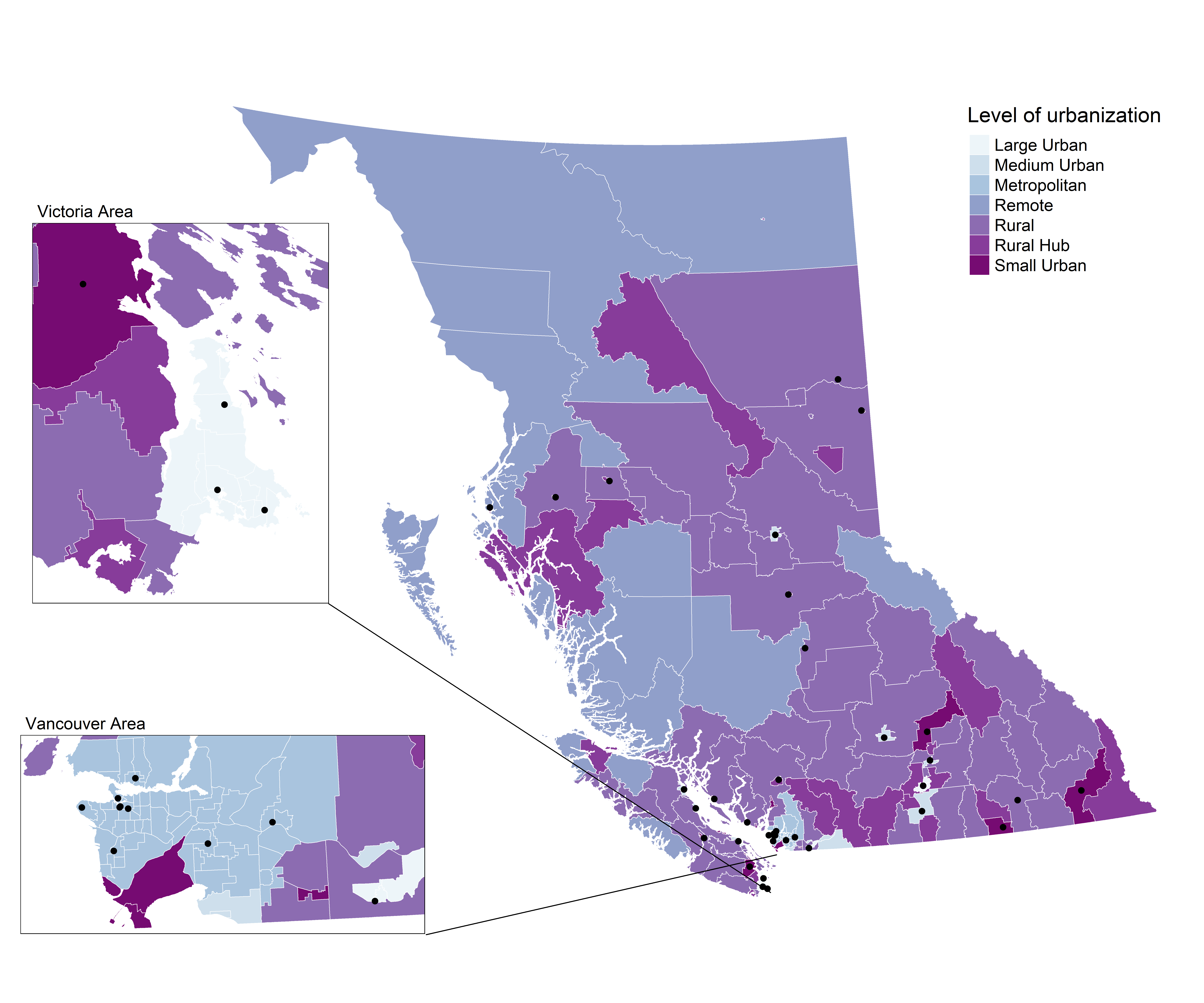

Supplement: Supplementary file 1 [file curroncol-30-00586-s001.zip › supp_fig_1.tiff]
